# Resource predictability modulates spatial-use networks in an endangered scavenger species

**DOI:** 10.1186/s40462-023-00383-4

**Published:** 2023-04-20

**Authors:** Catuxa Cerecedo-Iglesias, Frederic Bartumeus, Ainara Cortés-Avizanda, Joan Ll. Pretus, Antonio Hernández-Matías, Joan Real

**Affiliations:** 1 Equip de Biologia de la Conservació, Departament de Biologia Evolutiva, Ecologia i Ciències Ambientals, Facultat de Biologia and Institut de la Recerca de la Biodiversitat (IRBIO), Universitat de Barcelona, Diagonal 643, 08028 Barcelona, Catalonia Spain; 2 Centre d’Estudis Avançats de Blanes (CEAB), CSIC, Accés a la Cala Sant Francesc, 17300 Blanes, Girona, Spain; 3 Centre de Recerca Ecològica i Aplicacions Forestals, CREAF, Campus Bellaterra, 17300 Cerdanyola del Vallès, Spain; 4 Institució Catalana de Recerca i Estudis Avançats, ICREA, Passeig Lluís Companys 23, 08010 Barcelona, Spain; 5 Department of Plant Biology and Ecology, Faculty of Biology, University of Seville, Avda. Reina Mercedes 6, 41012 Seville, Spain

**Keywords:** Egyptian vulture, Foraging movements, Landfills, Predictable anthropogenic food subsidies (PAFS), Spatial networks, Space use, Spatial connectivity

## Abstract

**Background:**

Changes in human-induced resource availability can alter the behaviour of free-living species and affect their foraging strategies. The future European *Landfill Waste Directive* and *Circular Economy Action Plan* will reduce the number of predictable anthropogenic food subsidies (PAFS), above all, by closing landfills to preclude negative effects on human health. Obligate avian scavengers, the most threatened group of birds worldwide, are the most likely group of species that will be forced to change their behaviour and use of space in response to landfill site closures. Here, we examine the possible consequences of these management decisions on the foraging patterns of Egyptian vultures (*Neophron percnopterus*) in an expanding population in the Iberian Peninsula.

**Methods:**

We tracked 16 individuals in 2018–2021, including breeders and non-breeders, and, using a combination of spatial-use and spatial-network modelling, assessed landscape connectivity between key resources based on movement patterns. We then carried out simulations of future scenarios based on the loss of PAFS to predict likely changes in the movement patterns of both non-breeders and breeders.

**Results:**

Our results show that foraging strategies in non-breeders and breeders differ significantly: non-breeders performed more dispersal movements than breeding birds across a spatial-use network. Non-breeding and breeding networks were found to be vulnerable to the removal of central foraging areas containing landfill sites, a highly predictable resource, while perturbation analysis showed dissimilar foraging responses to the gradual reduction of other predictable resources. Under a context of the non-availability of landfills for breeders and non-breeders, vultures will increase their use of extensive livestock as a trophic resource.

**Conclusions:**

Future environmental policies should thus extend the areas used by scavengers in which livestock carcasses are allowed to remain in the wild, a strategy that will also mitigate the lack of food caused by any reduction in available waste if landfills close. In general, our results emphasize the capabilities of a spatial network approaches to address questions on movement ecology. They can be used to infer the behavioural response of animal species and, also demonstrate the importance of applying such approaches to endangered species conservation within a context of changing humanized scenarios.

**Supplementary Information:**

The online version contains supplementary material available at 10.1186/s40462-023-00383-4.

## Introduction

Many human activities result in modifications in both the spatial distribution and availability of trophic resources, thereby altering the behaviour of wildlife species [1–3]. Alterations of spatial-use strategies by individuals when exploiting resources (e.g. foraging [4, 5]) may ultimately determine the survival and reproductive performance of wildlife populations worldwide [6]. A better understanding of how species respond to human-induced changes in the availability of food resources is needed to (1) assess the expected effect of environmental policies on their food resources and (2) design conservation actions to counterbalance the negative effects of human-altered environments (see review [7]). Resource exploitation patterns in humanized environments are particularly worrying in the case of avian scavengers, for which available evidence indicates that predictable anthropogenic food subsidies (PAFS) may influence their use of space and movement patterns [8–10]. This avian guild includes vultures, one of the most world’s most endangered group of birds [11] and thus their conservation management is critical [12, 13].

The term PAFS refers to resources of anthropic origin whose appearance is predictable over space and/or time [9]. The most common example of PAFS are the landfills that have become an important predictable—and unlimited—source of food for many scavenger species, and the predominant food resource for many of them [5, 14–17]. Other example of PAFS are supplementary feeding stations, also known as ‘vulture restaurants’, where humans intentionally offer resources to wild scavengers as part of specific conservation measures or leisure activities (e.g., [18, 19]). The relative costs and benefits of PAFS use by scavengers are controversial because, while positive effects have been described in terms of breeding success [16, 20], the increase in the number of scavenger individuals in places with great food abundance can cause a density-dependent depression of productivity parameters [21]. In this paradoxical context, although vultures as obligate avian scavengers have evolved to depend on ephemeral and unpredictable carrion resources [22–24], the intensification of livestock farming practices and the increase in the number of PAFS may have led them to adapt their foraging strategies [25, 26], especially when their main food resources originate from landfills [15]. In Europe the availability of human waste as a feeding resource is expected to decrease drastically owing to the future *Landfill Waste Directive* (2008/98/EC) and the *Circular Economy Action Plan* [27], which contemplate the closure of landfills as a health-improving measure. Therefore, the study of the movement behaviour of vulture species in relation to trophic resources in European systems is an excellent scenario for understanding how birds exploit PAFS, as well as the effects they have on feeding resources due to the implementation of waste-management measures. In addition, more detailed research on how avian scavengers respond to this reduction in food availability is urgently required to shed light on management designed to preserve populations of some of the continent’s most endangered avian species.

Several approaches have been developed to study movement behaviour including state-of-the-art animal tracking by telemetry that can explore movements by wild animals [28–30]. The traditional approach to studying and analyzing animal movement with telemetry data uses kernel density estimators [31], which measure the intensity with which animals use different areas in their home ranges. A network approach has been used in ecological studies, above all to characterize food webs (e.g. [32]) and interactions between species (e.g., [33]). Yet, little attention has been paid to spatial ecology [34, 35], which focuses on the relationship between the environment and network topology. The spatial network approach using graph theory (see [36]) provides a graphic description of complex biological systems (e.g. composed of individuals) based on a set of nodes (i.e. areas with resources) interconnected by links (e.g. movement paths). Spatial networks can provide new insights into how animals interconnect in key areas (i.e. nodes) by movements (i.e. links between nodes) at landscape scale. In addition, using a novel network approach we can determine how the availability of PAFS influences vulture movement behaviour and so identify priority areas for conservation due to the strong spatial connectivity between key central areas [37]. In addition, by generating simulations based on variations in topological networks we can plausibly predict changes in spatial use caused by key alterations in spatial features (e.g. removal of well-connected nodes [35, 36]).

Here, we use spatial network analyses to investigate changes in movement behaviour in free-ranging Egyptian vultures (*Neophron percnopterus*) as responses to food availability. Firstly, we identified the key resources within home ranges and their connectivity at landscape scale (i.e. how animals forage between different food resources). Secondly, we tested the effect of different types of perturbations (i.e. resource-removal simulations) on resource prioritization and infer a population-level response.

The Egyptian vulture, an avian scavenger considered as ‘Endangered’ worldwide, has one of its strongest populations in the Iberian Peninsula, where its population trend is classified stable or slightly decreasing [38]. Here, we study a population in the northern Iberian Peninsula that over the past two decades has increased in size and even colonized new areas of a highly anthropogenically modified region [39, 40]. The individuals from this population exploit a wide range of food resources, ranging from small wild prey to large carcasses originating from extensive and intensive grazing regimes placed in ‘vulture restaurants’ [17], as well as resources obtained in large landfills [26, 39]. These sites exert an important attraction during the exploration and exploitation movements of these vultures [39, 41–43]. Thus, we used a network approach to (1) examine the foraging behaviour and spatial-use patterns of Egyptian vultures in an anthropogenically modified landscape; and (2) to predict individual foraging responses to the reduction and/or closure of PAFS. In addition, we addressed certain research and conservation measures in light of the future circular economy scenarios. Our initial hypotheses were that focal non-breeders and breeders would have different foraging strategies due to distinct spatial networks, and that the elimination of PAFS nodes would have a differential impact on non-breeders and breeders. We predicted that non-breeders, which are not tied to a particular breeding site, would have larger home ranges with a significant number of nodes of highly predictable feeding sites and would be seriously affected by the closure of PAFS, while breeders, which are tied to a nest site, would be more likely to exploit unpredictable food resources at fewer sites and be less influenced by landfill closures. Consequently, different conservation strategies are required for these two types of vulture populations.

## Methods

### Data collection

We tagged 16 breeding Egyptian vultures—six breeding adults (i.e. 5 year-old or older) and 10 non-breeders (1 adult and 9 immatures)—with GPS-GSM devices during the summers of 2018 and 2019. All birds were captured at a landfill site in Osona (Catalonia, Spain). We equipped eight birds with 40-g solar-powered e-Obs satellite transmitters GPS-GSM (www.e-obs.de) and eight birds with Ornitela (www.ornitela.com) digital telemetry devices using a Teflon ribbon harness. Captured birds were aged according to plumage (Additional file 1: Table S1; [44], pers. data). As we were only interested in studying movements during the summer, we discarded migration locations and winter quarters from the data. We considered the beginning of the breeding period to occur when the rectilinear migration path of individuals from Africa began to show great sinuosity on arrival in the study area, and the end of the period when we began to observe, conversely, a rectilinear southwards path. We were interested in prospecting and feeding behaviour during the day and so to optimize the energy performance of the devices the sleep interval of the e-Obs tags was set as 18 h ON/6 h OFF (6:30–22:30, Coordinated Universal Time) and for the Ornitela tags set in terms of the relative 18° sun angle above or below the horizon. We scheduled the GPS devices to record one location every 30 min and, as we were focused on foraging behaviour, we only selected locations within the daily time intervals between sunrise and sunset where birds were active. Paired individuals in adult plumage holding a breeding territory were classified as breeders whilst nomadic individuals not linked to a breeding territory were classified as non-breeders [45].

### Spatial-use networks based on landscape features

We built two spatial networks based on the reproductive status of birds (non-breeders vs. breeders) composed of nodes and links to determine how animals interconnect feeding areas along movement paths. The nodes—the areas most used by all individuals—were spatially delimitated as follows. First, we measured the home range of all tagged individuals using the 50% Dynamic Brownian Bridge Movement Model (dBBMM) for each individual and year to represent the core areas in which these birds spent the most time. The dBBMM algorithm allows us to estimate the spatial-use likelihood by taking into account the temporal dependency of GPS data. The outcome of the dBBMM algorithm is a probability layer with a 500-m^2^ grid cell known as the Utilization Distribution (UD [46]), a probability that refers to the likelihood of a specific area being used by an individual or individuals. Then, we calculated UDs at individual-year level by averaging all UDs to obtain a single global home range that clearly defines all the available geographical areas that any of the birds would use (Additional file 1: Fig. S1). Second, given that our home range at 50% contour at population level was composed of several polygons, each was considered to be a node. The links were the movement paths (i.e. movements of animals between nodes) that focal vultures performed when connecting a ‘departure’ node to an ‘arrival’ node. The frequency of the links between two nodes equated to the strength of the spatial connection. As we found very few movement paths between nodes with a duration of less than half an hour (less than 10% of the trips connecting two nodes), we selected only movement paths lasting one hour or more.

To analyse which environmental factors influenced the movement paths and space used in the networks, we characterized the nodes with nine land-cover categories taken from the CORINE 2018 Land Cover (www.land.copernicus.eu/) program (see Table 1) and with three ecological categories: feeding, roosting or breeding territories (the latter only for breeding birds). For feeding, we considered five types of food resources: landfill sites, intensive farms, vulture restaurants, extensive livestock, and other unpredictable resources (ordered by predictability over time and spatial heterogeneity). In addition, we selected the main food resource of each node by overlapping the UD layer of all tagged individuals and the resource location layer using both the CORINE Land Cover layer and satellite images. We assigned one resource type to each node by selecting the food source with the greatest probability of use according to the UD values. Roosting sites and breeding territories are binary features indicating whether or not a roosting site or breeding territory is present within the node (Additional file 1: Table S2). Roosting sites are communal roosts where birds socialize. To verify breeding territories, i.e. the areas where breeding individuals build their nests, at least one visit to the breeding territory was made between April and July.

**Table 1:** Description of the metrics of the networks (A) and the features of the nodes (B) for the non-breeders and breeders’ Egyptian vultures in the study area

(A) *Network metrics*	Level	Description
*Diameter*	Network	The length (in number of edges) of the longest path through the network from one node to another between any two vertices
*Density*	Network	The average probability that two nodes that are network neighbours are themselves neighbours of another node
*Degree*	Nodes	The number of links joining a node to its neighbours
*Betweenness*	Nodes	The number of shortest paths through the network from one node to another that passes through a given node (the highest values are also called hubs)

(B) *Nodes features*	Classes (Acronym)	
Land-use (Acronym)	Forest (FOR)	Cover (%) of forest per node
	Pastureland (PAS)	Cover (%) of pastureland per node
	Scrubs (SCR)	Cover (%) of scrubs per node
	Irrigated crops (IRR)	Cover (%) of irrigated crops per node (e.g., rice)
	Non-irrigated crops (NIC)	Cover (%) of non-irrigated crops per node (e.g., wheat)
	Permanent crops (TREE)	Cover (%) of permanent crops per node (e.g., olives)
	Bare rock (ROC)	Cover (%) of bare rock per node
	Urban areas (URB)	Cover (%) of urban areas per node
	Others (OTH)	Cover (%) of other typologies of land uses per node
Ecological functions	Resources	Set of food sources (categorized in: landfills, extensive and intensive farms, and vultures’ restaurant
	Resting	When a node is used as roosting site
	Breeding territory^a^	When a node has a known nest

^a^Only for breeders

We characterized the network topology using two quantitative metrics at network level (*diameter* and *density*) and two quantitative metrics at node level (*degree* and *betweenness*; see Table 1 and Additional file 2). Metrics at network level describe on average the movement paths of focal birds. To measure the average length of movement paths (i.e. the movement between nodes or links), we calculated the *diameter*, which reflects the speed of movement through a network and scales up as more nodes are used by the focal birds. Therefore, a larger *diameter* implies a greater dispersing capacity in the focal birds, while *density* measures the heterogeneity of the averaged movement paths. The heterogeneity shows how movement paths and space use differ during an individual's movements inside the network. Homogeneous networks (lower *density* values) have the same number (on average) of links per node, whereas heterogeneous networks (greater *density* values) differ in the number of links per node [47]. In biological terms, *density* illustrates whether the movement paths of birds are random or non-random [35]. Metrics at node level indicate the relative importance of a node in terms of connectivity and show the core locations to which animals are attracted. Thus, by measuring the number of links of each node in terms of its interaction with neighbourhood nodes, we calculated the *degree* to identify which nodes were most heavily used by individuals. We used *betweenness* to measure the frequency of a node as an intermediate step between the path of two other nodes. Higher values of *betweenness* represent a more central position for nodes with large numbers of links to other nodes (i.e. connectivity: [47]). The nodes with the highest values of *betweenness—*known as hubs—were considered to have greater relative importance in the foraging movements of individual birds [48].

Finally, we calculated two sets of parameters: first, the node fidelity was used to understand in detail the effects of node features on the use of space and was defined by (1) the number of revisits that individuals make to a specific node and (2) the accumulated residence time that focal birds spent at each node. Second, the spatial connectivity of non-breeders and breeders was represented by the *degree* and *betweenness*.

### Statistical analyses

We used the F-statistic of analysis of variance (ANOVA) to test differences between non-breeders and breeders in the metric parameters of their foraging behaviour at network level. We compared the number of elements (nodes and links) and the network quantitative metrics at network level (*diameter* and *density*) in terms of reproductive status [49]. We performed linear regressions to identify which features of the nodes determined node fidelity (number of revisits and residence time) and node importance in terms of interconnections along movement paths (*degree* and *betweenness*). To do this, we fitted separate models for each response variable (number of revisits, residence time, *degree* and *betweenness*) and each reproductive status because the spatial-use networks of non-breeders and breeders were completely different (see Results). For each model, we estimated the importance of each explanatory variable (node features described above) by removing it from the model and then performing an F-ratio test to derive *P* values for the variable of interest [50]. In terms of ecological functions, nodes with breeding territories were only considered in the linear model for breeders. In order to avoid collinearity between each category of land cover in the linear regressions, we carried out a Principal Component Analysis (PCAs) to reduce the number of correlated explanatory variables to just a few uncorrelated variables (orthogonal). Each Principal Component (PC) was obtained from the covariance matrix of the original variables [49].

Finally, we used a perturbation analysis to assess the foraging responses of individuals under future scenarios linked to the limitation of PAFS by environmental regulations. We simulated different perturbations on the network by removing nodes of different types and computing network robustness and the presence of key nodes according to available food resources. Network robustness refers to the ability of a network to maintain its features regardless of the degradation of the network itself [48]. The structure of spatial-use networks is characterized by their elements and their distribution as any degradation of their structure may modify the movement paths of individuals and reveal the underlying robustness (or vulnerability) of the connection (or disconnection) between key areas [48]. So, we first performed a ‘random removal’ of nodes and the subsequent measures of *betweenness* at each iteration. We then performed ‘targeted removal’ by removing nodes with a specific feature (e.g. nodes where landfills are present) until there were no more nodes with a specific feature, and then recalculated the *betweenness* measures for each iteration. In both cases, each iteration refers to the gradual one-by-one removal of nodes. ‘Random removal’ of nodes allowed us to infer whether the foraging response to the limitation on PAFS is a stochastic process if compared to the ‘targeted removal’ of nodes, or whether it follows a deterministic process. Therefore, we ran each random and targeted removal iteration 1000 times to generate two different frequency histograms of the *betweenness*. Finally, we compared these histograms resulting from each random and targeted simulation using a paired T-test. In such a way, we identified which node feature drives the robustness of both the non-breeder and breeder spatial-use networks.

All analytical procedures were carried out within the R environment [51] using the *recurse* [52], *move* [53] and *igraph* packages [54].

## Results

**Fig. 1:**
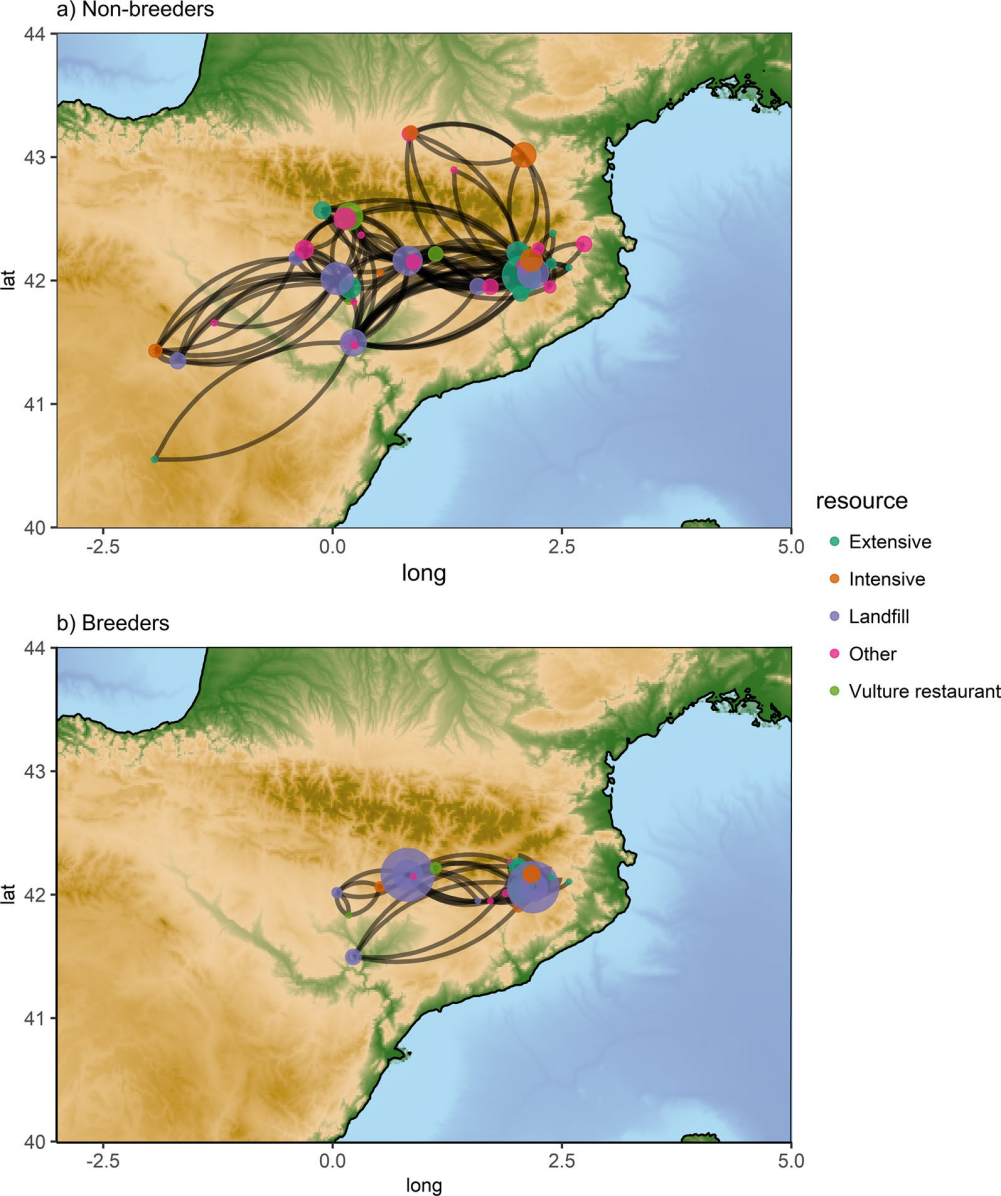
Spatial-use networks of **a** non-breeding and **b** breeding Egyptian vulture populations in the study area. At the nodes the type of resources (i.e. landfill, intensive farm, extensive farm, and vulture restaurant and others that act as random or ephemeral resources) are shown. Node sizes are proportional to the *betweenness* value. Links represent the foraging trips connecting nodes. The length of the links depends on the frequency of the movement paths between the two nodes

When considering all individuals, i.e. non-breeders and breeders, the spatial-use network included a total of 44 nodes scattered throughout the northeast Iberian Peninsula (Catalonia, Aragón, Navarra, and Castilla y Leon) and southern France (Additional file 1: Fig. S1). Non-breeding and breeding vultures had different patterns of space use, as illustrated by the ANOVA in the topology of the spatial-use network. For example, the number of nodes (mean ± SD; non-breeders 11.7 ± 2.31; breeders 3.5 ± 1.38) and links (mean ± SD; non-breeders 38.3 ± 10.4; breeders 7.17 ± 5.53) were significatively higher in the networks of non-breeders than in breeders (nodes: F_4,600_ = 61.29, *P* < 0.001; links: F_4,600_ = 45.16, *P* < 0.001). Moreover, comparing the *diameter* and *density* at network level between non-breeders and breeders, we found two completely different patterns of movement. Compared to breeders, non-breeders had more dispersive movement paths (Mean ± SD; non-breeders 5.4 ± 1.65; breeders 2.33 ± 1.03; F_4,600_ = 16.61, *P* < 0.01) and made many more random and heterogeneous movements (mean ± SD; non-breeders 0.04 ± 0.01; breeders 0.008 ± 0.006; F_4,600_ = 45.16, *P* < 0.001). Nevertheless, for both breeders and non-breeders landfills were central areas during their movements. Nodes in which landfills were present had the highest *betweenness* and acted as hubs connecting the other nodes with different space use and ecological features (Fig. 1).

In the non-breeder spatial-use network, multiple linear regression analysis revealed that node fidelity (number of revisits and residence time) was related to the presence of roosting sites, extensive livestock and intensive farms and landfills (revisits: F_5,35_ = 6.61, *P* < 0.001, R^2^ = 0.49; residence time: F_6,34_ = 10.7, *P* < 0.001, R^2^ = 0.65; Table 2); however, land-cover classes were not good predictors for explaining node fidelity or the relative importance of nodes within the spatial-use network (Additional file 3: Table S4). We selected two PCs in non-breeder regressions that explained a total of 75% variance. We found that PC1 relies positively on forest and negatively on non-irrigated land cover (Additional file 1: Table S3). Non-irrigated crops and forest (i.e. PC1; Additional file 1: Table S3) were selected to explain residence time but had no significant effect on non-breeders’ use of networks. Moreover, the nodes most used by non-breeders were explained by roosting sites and the presence of landfills and extensive livestock (*degree*: F_5,35_ = 9.29, *P* < 0.001, R^2^ = 0.57), both factors having a positive effect on the *degree.* Likewise, the nodes considered as central areas were positively driven by the presence of landfills (*betweenness*: F_4,35_ = 5.4, *P* > 0.05, R^2^ = 0.38). On the other hand, for breeding individuals, node fidelity was positively explained by breeding territory (number of revisits: F_5,13_ = 5.53, *P* < 0.05, R^2^ = 0.68) and extensive livestock (residence time: F_4,14_ = 4.4, *P* < 0.05, R^2^ = 0.56; Table 3). However, no significant effect of node features was found to explain *degree* and *betweenness* in the spatial-use networks of breeding birds (see simple ANOVA comparisons in Additional file 1: Figs. S3 and S4).

**Table 2:** Multiple linear regressions analysis for site fidelity (number of revisits and residence time) and network connectivity (*degree* and *betweenness*) of non-breeder Egyptian vultures

	Non-breeders																			
	log(number of revisits)					log(residence time)					log(degree)					*Betweenness*				
Variables	*E*	*SE*	95% CI		*P*	*E*	*SE*	95% CI		*P*	*E*	*SE*	95% CI		*P*	*E*	*SE*	95% CI		*P*
			*LL*	UL				*LL*	UL				*LL*	UL				*LL*	UL	
(Intercept)	0.84	0.37	0–09	1.59	**0.029**	0.64	0.59	− 0.57	1.84	0.291	1.15	0.17	0.80	1.50	**< 0.001**	29.48	1.43	− 12.50	71.46	0.163
PC1	–	–	–	–	–	− 0.18	0.10	− 0.38	0.02	0.082	–	–	–	–	–	–	–	–	–	–
Resources																				
Extensive	0.91	0.44	0.02	1.79	**0.046**	1.48	0.71	0.04	2.92	**0.044**	0.52	0.21	0.09	0.96	**0.020**	14.68	0.43	− 55.28	84.64	0.673
Intensive	1.80	0.55	0.68	2.92	**0.003**	2.01	0.90	0.17	3.84	**0.033**	0.55	0.27	0.00	1.09	0.052	48.53	1.15	− 37.50	134.55	0.260
Landfill	2.09	0.51	1.05	3.14	**< 0.001**	2.51	0.87	0.75	4.27	**0.006**	1.01	0.25	0.49	1.52	**< 0.001**	167.98	4.48	91.90	244.07	**< 0.001**
Vulture rest	0.72	0.69	− 0.69	2.13	0.310	1.18	1.14	− 1.13	3.49	0.307	0.35	0.34	− 0.34	1.05	0.312	64.19	1.23	− 41.45	169.83	0.226
Roosting																				
YES	1.59	0.41	0.77	2.42	**< 0.001**	3.87	0.73	2.40	5.35	**< 0.001**	0.73	0.20	0.34	1.13	**< 0.001**	–	–	–	–	–
					*R*^*2*^ = .595					*R*^*2*^ = .654					*R*^*2*^ = .570					*R*^*2*^ = .381

Significant *P* values < 0.05 are in bold

*E* estimate, *SE* standard error, *CI* confidence interval, *LL* lower bound at 95% level of confidence, *UL* upper bound at 95% level of confidence, *P *values. R^2^ represent the coefficient of determination for each selected model which does not include all explanatory variables

**Table 3:** Multiple linear regressions analysis for site fidelity (number of revisits and residence time) breeder Egyptian vultures

	Breeders																			
	log(number of revisits)					log(residence time)					log(degree)					*Betweenness*				
Variables	*E*	*SE*	95% CI		*P*	*E*	*SE*	95% CI		*P*	*E*	*SE*	95% CI		*P*	*E*	*SE*	95% CI		*P*
			*LL*	UL				*LL*	UL				*LL*	UL				*LL*	UL	
(Intercept)	1.33	0.60	0.04	2.62	**0.045**	1.93	1.16	− 0.56	4.42	0.119	1.68	0.17	3.21	2.11	**< 0.001**	14.59	5.42	3.21	25.98	**0.015**
Resources																				
Extensive	2.34	1.08	0.00	4.68	0.050	5.98	1.74	2.25	9.71	**0.004**	–	–	–	–	–	–	–	–	–	–
Intensive	− 0.10	1.01	− 2.28	2.09	0.924	1.16	1.90	− 2.91	5.22	0.552	–	–	–	–	–	–	–	–	–	–
Landfill	0.99	0.86	− 0.86	2.85	0.269	3.38	1.64	− 0.14	6.90	0.058	–	–	–	–	–	–	–	–	–	–
Vulture rest	− 1.36	1.19	− 3.93	1.20	0.272	− 1.48	2.17	− 6.13	3.18	0.508	–	–	–	–	–	–	–	–	–	–
Breedeing territory																				
YES	1.86	0.81	0.12	3.61	**0.038**	–	–	–	–	–	–	–	–	–	–	–	–	–	–	–
					*R*^2^ = .680					*R*^2^ = .557										

Significant *P* values < 0.05 are in bold

*E* estimate, *SE* standard error, *CI* confidence interval, *LL* lower bound at 95% level of confidence, *UL* upper bound at 95% level of confidence, *P* values. R^2^ represent the coefficient of determination for each selected model which does not include all explanatory variables. Models related to spatial connectivity (*degree* and *betweenness*) are not significant (null models are shown)

**Fig. 2:**
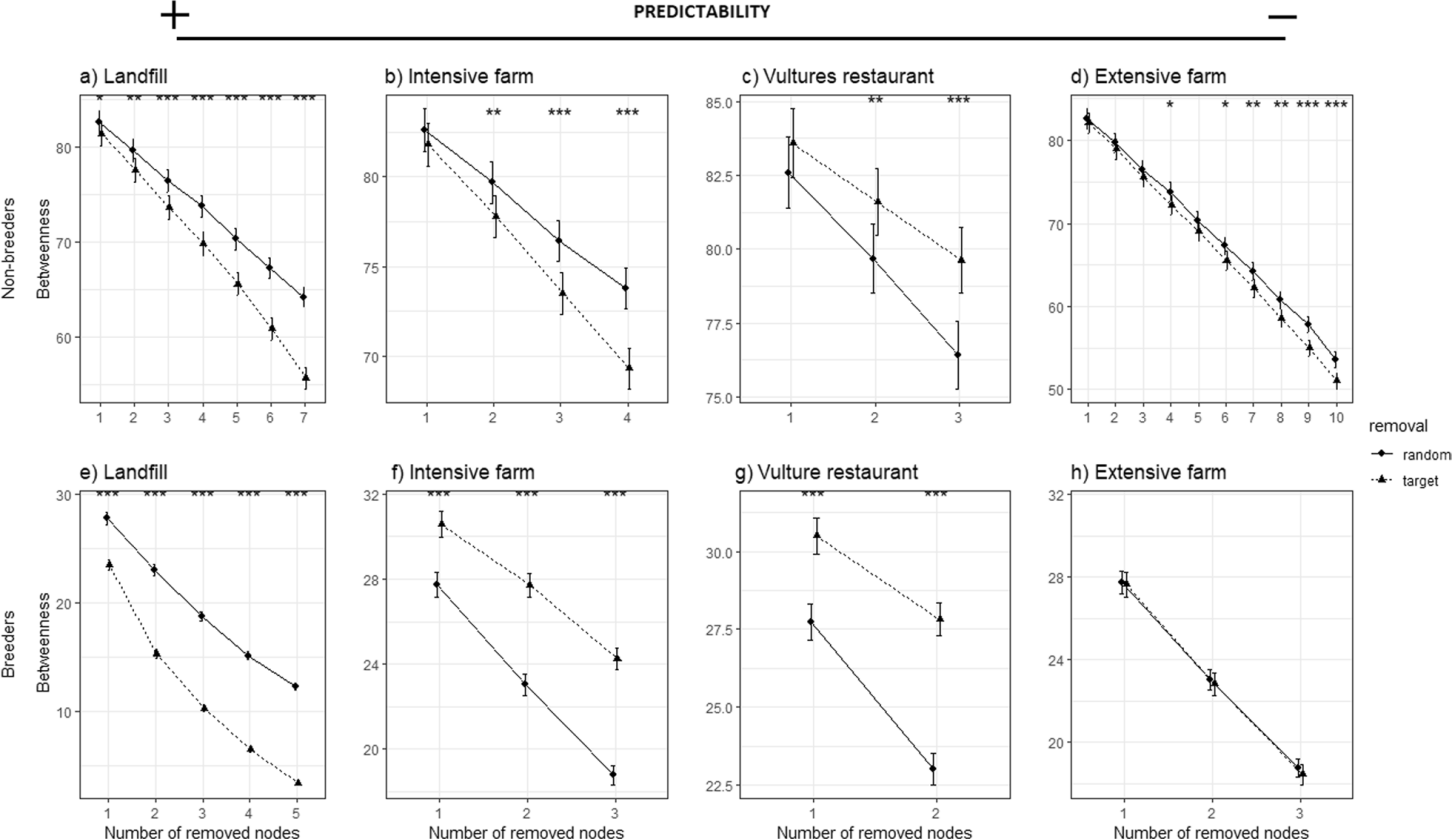
Environmental perturbation analysis. Random and Targeted node removal used to examine the response of non-breeding (**a**–**d**) and breeding (**e**–**h**) Egyptian vultures to future sanitary legislation if implemented. Plots show the mean *betweenness* values and confidence intervals along, respectively, random (circle or thick line) or targeted (triangle or dashed line) node-removal simulations. Coefficient intervals (5–95%) are shown. Aesthetics show significant differences (*P < 0.05; **P < 0.01; ***P < 0.001)

In general, the perturbation analysis showed that the gradual disappearance of PAFS will significantly alter the movement paths and the degree of relative importance of nodes (connectedness of nodes) and, in turn, modify the foraging strategy of these two subsets of this vulture population (*P* values for paired T-tests comparing random and targeted simulation were less than 0.05; Fig. 2). The removal of key nodes where landfills exist would have a deterministic effect on foraging movements at population level resulting in an increase in nodes that are poorly connected to the non-breeding and breeding networks, as shown by the significantly lower *betweenness* values of the targeted simulation compared to the random simulation (Fig. 2a, e). Moreover, the removal of nodes from the non-breeding network, where intensive or extensive farms are the main food resource, would have a similar impact on foraging strategies as landfill-site removal but at a lower intensity (Fig. 2b, d). Thus, the non-breeding network will be slightly more robust in the event of the disappearance of intensive and extensive farms. Compared to the non-breeding network, the breeding network was found to be more resilient to intensive farm removal as key nodes would become either more interconnected or there would be an increase in the number of well-connected nodes (Fig. 2f). Similar foraging responses in both non-breeding and breeding networks were found when nodes with vulture restaurants were removed (Fig. 2c, g). In addition, the perturbation analysis showed that there were no effects on foraging responses when we removed nodes with extensive farms from the breeding network (Fig. 2h).

## Discussion

It is well-known that transformations of human-mediated ecosystems have the potential to alter animal movement patterns and foraging behaviour (e.g. [5, 55–57]). By studying changes in space use and connectivity in an endangered vulture species, the Egyptian vulture, we improved our understanding of how environmental and ecological conditions influence the foraging movements of different fractions of the population (i.e. non-breeders and breeders) in different ways. We show here that landfills are a key environmental factor driving spatial-use patterns and how inferred future scenarios in the event of landfill closure will generate profound changes in movement patterns in terms of connectivity in this endangered vulture’s populations.

Non-breeding and breeding vultures have different foraging strategies, as illustrated by the differences in the spatial topology of their networks. Non-breeding individuals disperse more along a spatial-use network that has more nodes and links than the breeding bird networks. This could be related to the larger exploratory capacity of non-breeders as they have no nest attachments or functional constraints imposed by the demands of breeding. It is known that non-breeders have larger home ranges than breeders [42, 43] and this is probably associated with the greater number of areas they visit and more connections between them (networks with more nodes and links). The exploratory foraging behaviour undertaken by non-breeders may also explain the heterogeneity of movements found in their networks, in which individuals rarely use or connect certain spatial areas and mostly travel through well-used and well-connected areas, the so-called hub-nodes. Such heterogeneous topologies are reminiscent of the limiting case of scale-free network properties (see [58]; Additional file 4). This special type of network has been described in other species (e.g. bats, [37]) and are known to be robust against random-node removal but susceptible to (hub)-node removal [59]. In the foraging networks of non-breeding Egyptian vultures, hub-nodes are represented by landfills and intensive farms. Thus, it is not surprising that the main roosting sites in our study area are near landfills (pers. obs.), which are closely associated with predictable food sources. These roosting sites are both stopovers during migration and temporary settlement areas during the breeding season where individuals socialize and exchange information [60–62]. As well, landfills may act as highly visible and familiar landmarks or waypoints along movement paths that aid navigation between other nodes, a mechanism that has been reported in Western Gulls (*Larus occidentalis*) [57]. Conversely, the territorial behaviour of breeders is characterized by low dispersal and homogeneous movements, and individuals travel between nodes with a similar degree of usability (exploitability) and connectivity. We found a parallel in seabird literature, in which researchers also described generally more specialized foraging behaviour in breeding adult Northern Gannets (*Morus bassanus*) than in non-breeding birds, almost certainly imposed by their central foraging behaviour and habitat use [63]. The space use and connectivity emerging in breeding individuals is thus potentially vulnerable to random landscape transformation but less sensitive to targeted landscape transformations. This is probably due to the few nodes present in breeders’ spatial-use networks, in which the slightest alteration spreads quickly and has a strong effect on network topology, as has been described in other kinds of networks [64]. Although the increase in our focal population over the past decades is probably linked to the appearance of landfills [26], it is known that extensive livestock can also act as one of the main food sources in breeding territories far from landfills [17]. Accordingly, our results support the idea that breeding birds are currently heavily reliant on extensive livestock [17, 65]. Overall, our findings agree with past studies regarding the interconnection between space use and reproductive status in vultures [66–68] despite our use of a novel application of a network approach to shed light on movement patterns during foraging, and use of the connectivity between distinct feeding resources in two subsets of an Egyptian vulture population. We also demonstrate here that predictable food availability affects large-scale movement behaviour in avian scavengers, as has been recognized in other species (e.g. seabirds [55]; white storks [5]; brown bears [56]; gulls [16]).

**Fig. 3:**
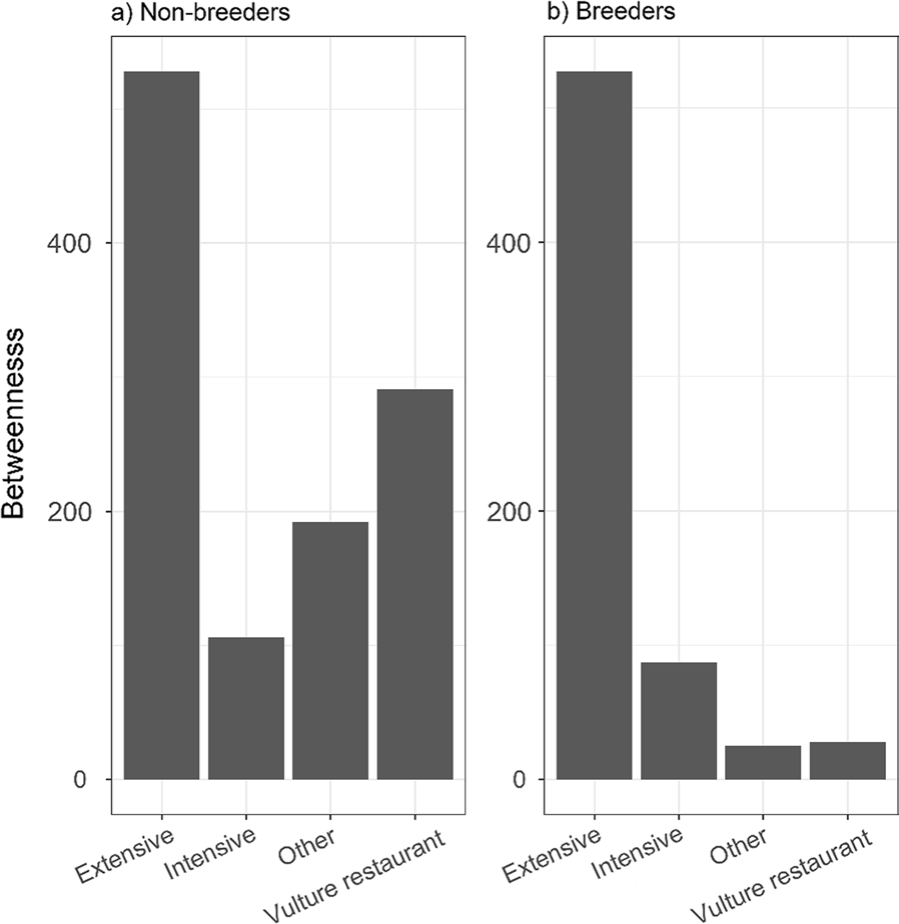
Average *betweenness* of spatial-use networks for **a** non-breeding and **b** breeding Egyptian vulture populations in terms of the different sets of resources for all nodes from which landfills were removed

Perturbation analysis demonstrates that both non-breeder and breeder foraging strategies are vulnerable to the removal of nodes with highly predictable food sources, especially if landfills are present. Our results show that the systematic removal of landfills (hub-nodes) changes patterns of population movements such that other nodes become key in the use-of-space strategies of focal birds. Therefore, when a node with a landfill is removed, another node with a different food resource becomes the new hub. In line with our prediction, we found that this is especially important for non-breeding birds whose movements mainly target local areas with landfills (see also [43]). A possible explanation for this foraging pattern could be their lack of experience in prospecting, as well as their lack of dependency on a breeding territory, which favours the exploitation of predictable food sources (see e.g. [69]). By contrast, although we predicted that the disappearance of PAFS would not affect the foraging behaviour of breeding individuals, interestingly our results did show that an important alteration in movement patterns occurred if landfills, intensive farms and vulture restaurants were sequentially removed. The fact that breeders take advantage of less predictable resources such as extensive livestock is most likely due to their location near breeding territories [17]. Indeed, at population level, our simulations predict that, in the lack of landfill-site scenario, the core behavioural response of birds will be to switch to extensive livestock (see Fig. 3), which thus makes extensive livestock a key element in any future conservation strategy.

The studied Egyptian vulture population showed a great dependence on PAFS and our results indicate that the future closure of landfills (see 2008/98/EC [27]) will reconfigure their spatial networks and lead to a shift in home ranges in such a way that landfills will no longer be their central foraging areas. Currently, landfills concentrate large numbers of individuals of different ages and reproductive status from both local and neighbouring populations (e.g. France, Spain; pers. obs.). This makes them key places for information exchange, socialization and roosting, as well as a profitable feeding sites, particularly for non-breeders (e.g. [70]), and, in turn, for the recruitment and viability of local and regional populations ([26]; pers. obs.). An option for filling this gap in food provision if landfills close is to favour a natural supply of carrion, if necessary, by maintaining certain supplementary feeding points specifically targeting Egyptian vultures and non-breeder survival ([71, 72]) and/or guaranteeing the connection between non-breeding and breeding populations that ensures population viability. In fact, some vulture restaurants designed specifically for Egyptian vultures replace the roosting functions that landfills currently perform ([61]; pers. obs.). In any case, our findings suggest that more research is required into how PAFS affect the non-breeder subset of vultures. It is not clear to what extent landfill closure will affect the performance of breeding birds, although it is known that the occupancy of breeding areas is somehow related to these feeding sites [26]. Our results reveal that the spatial-use network of breeders is shaped above all by extensive farming and the benefits of this type of animal husbandry for vulture breeding populations have been noted elsewhere [17, 73]. Thus, future conservation farming policies should promote extensive livestock practices and allow more farmers to freely abandon livestock carcasses in the field. To do so, regional policies should focus on extending the areas in which the abandoning of extensive carcasses is permitted (e.g. in Spain, ZPAEN). Long-term monitoring is key to identifying how population numbers vary over time, and the combination of telemetric information and other tracking methods (e.g. ringing) will allow us to measure vital parameters and evaluate population viabilities under new food availability scenarios. In conclusion, we emphasize how movement ecology and network modelling are highly promising tools and can potentially play a key role in movement research. They allow us to predict the responses of wild species having to face up to environmental changes and landscape transformation (e.g. [33, 37]) and so will play a crucial role in the search for the most efficient conservation practices.

## Supplementary Information

**Additional file 1:** **Table S1**. Egyptian vultures tagged with GPS-GMS devices during the summer period between 2018 and 2019 in Catalonia (NE Spain). **Table S2**. Total of nodes used by non-breeder and breeder Egyptian vultures. **Table S3** Factor loadings after Principal Component Analysis for the nine categories assigned to land uses in the study area. **Figure S1**. Dynamic Brownian Bridge Models home ranges at 50% (dark grey) and 95% (light grey) of 10 non-breeders and 6 breeders of Egyptian vulture tagged in Catalonia (Northeast Spain) at the population level. **Figure S2**. Correlation between nodes’ features and the parameters related to node fidelity (number of revisits and accumulated residence time) and local network metrics (degree and betweenness) for the focal a) non-breeders and b) breeders. **Figure S3**. Boxplots of non-breeders node fidelity. **Figure S4**. Boxplot of breeders node fidelity.

**Additional file 2:** Topology parameters of spatial networks.

**Additional file 3:** **Table S4**. Results of the top-ranked models (lowest AIC) for non-breeder and breeder Egyptian vultures accounting for site fidelity (number of revisits and accumulated residence time) and spatial-use network topology (*degree* and* betweenness*).

**Additional file 4:** **Figure S5**.* Degree* (K) and* betweenness* (B) distributions of spatial-use networks for breeding and non-breeding populations of Egyptian vultures.

## Data Availability

The datasets generated and analyzed during the current study are not publicly available due to sensitive information on a threatened and endangered species. Data are however available from the authors upon reasonable request and with permission of contact person of the ‘*Neophron percnopterus* - Central Catalonia’ repository on Movebank (www.movebank.org).
