# Supplementary material for: Resource predictability modulates spatial-use networks in an endangered scavenger species
Source: Mov Ecol. 2023 Apr 20;11:22. doi: 10.1186/s40462-023-00383-4 (PMC10120099; doi:10.1186/s40462-023-00383-4)
Supplement: Supplementary file 1 — Additional file 1: Table S1. Egyptian vultures tagged with GPS-GMS devices during the summer period between 2018 and 2019 in Catalonia (NE Spain). Table S2. Total of nodes used by non-breeder and breeder Egyptian vultures. Table S3 Factor loadings after Principal Component Analysis for the nine categories assigned to land uses in the study area. Figure S1. Dynamic Brownian Bridge Models home ranges at 50% (dark grey) and 95% (light grey) of 10 non-breeders and 6 breeders of Egyptian vulture tagged in Catalonia (Northeast Spain) at the population level. Figure S2. Correlation between nodes’ features and the parameters related to node fidelity (number of revisits and accumulated residence time) and local network metrics (degree and betweenness) for the focal a) non-breeders and b) breeders. Figure S3. Boxplots of non-breeders node fidelity. Figure S4. Boxplot of breeders node fidelity. [file 40462_2023_383_MOESM1_ESM.docx]

**Additional file 1. Data available and exploration analysis**

**Table S1.** Egyptian vultures tagged with GPS-GMS devices during the summer period between 2018 and 2019 in Catalonia (NE Spain). Note that age (adults, A; immatures, I) was determined by plumage according to Blasco-Zumeta and Jeinze *et al.*, 2006 and breeding status (breeder, B; non-breeder, NB) was determined based on the GPS location and the monitoring of focal species. There were no changes in reproductive status at the individual level during the study period. Nodes visited per year and home rage (dBBMM) are also shown for each bird.

| ID | Tag devices | Reproductive status | Age class | Nodes visited | | | | Years tracked | dBBMM (km^2^) | |
| --- | --- | --- | --- | --- | --- | --- | --- | --- | --- | --- |
|  |  |  |  | 2019 | 2020 | 2021 | All years |  | 50% | 95% |
| 6977 | e-Obs | B | A | - | 3 | 4 | 4 | 2 | 21 (1) | 278 (22) |
| 6978 | e-Obs | B | A | - | 5 | 6 | 6 | 2 | 73 (11) | 735 (1) |
| 6616 | e-Obs | B | A | - | 3 | 2 | 3 | 2 | 22 (3) | 221 (49) |
| 181648 | Ornitela | B | A | - | - | 4 | 4 | 1 | 60 | 503 |
| 201397 | Ornitela | B | A | - | - | 3 | 3 | 1 | 58 | 576 |
| 200666 | Ornitela | B | A | - | - | 1 | 1 | 1 | 23 | 226 |
| 201395 | Ornitela | NB | A | - | - | 12 | 12 | 1 | 87 | 5613 |
| 6223 | e-Obs | NB | A | - | 8 | 5 | 8 | 2 | 50 (13) | 4632 (130) |
| 6979 | e-Obs | NB | I | - | 14 | 7 | 15 | 2 | 24 (10) | 2952 (267) |
| 6224 | e-Obs | NB | I | 10 | 10 | - | 14 | 2 | 137 (4) | 3155 (237) |
| 201398 | Ornitela | NB | I | - | - | 14 | 14 | 1 | 107 | 9412 |
| 6222 | e-Obs | NB | I | 10 | 8 | 10 | 13 | 3 | 151 (91) | 5129 (1395) |
| 6615 | e-Obs | NB | I | - | 10 | 10 | 13 | 2 | 128 (101) | 6857 (985) |
| 181649 | Ornitela | NB | I | - | 9 | 7 | 10 | 2 | 68 (7) | 2942 (992) |
| 201452 | Ornitela | NB | I | - | - | 10 | 10 | 1 | 12 | 1412 |
| 201399 | Ornitela | NB | I | - | - | 9 | 9 | 1 | 174 | 138835 |

Numbers inside parentheses are SD.

**Table S2.** Total of nodes used by non-breeder and breeder Egyptian vultures. The nodes are classified according to the following 3 ecological features: feeding, roosting, and breeding territory. We considered five food resources: landfills, intensive farms, vulture restaurants, extensive livestock, and other unpredictable resources. Roosting and breeding territory are binary features; YES implies that a roosting site or breeding territory is present within the node and vice versa.

| Ecological features | Level | N. of nodes used | |
| --- | --- | --- | --- |
|  |  | Non-breeders | Breeders |
| Feeding | Landfill | 7 | 5 |
|  | Intensive farm | 5 | 3 |
|  | Vultures’ restaurant | 3 | 2 |
|  | Extensive livestock | 10 | 4 |
|  | Other | 16 | 5 |
| Roosting | Yes | 29 | 16 |
|  | No | 12 | 3 |
| Breeding territory^a^ | Yes | 7 | 6 |
|  | No | 34 | 13 |
| Total |  | 41 | 19 |

^a^Only for breeders

**Table S3.** Factor loadings after Principal Component Analysis for the nine categories assigned to land uses in the study area.

| Land uses | Non-breeders | |  | Breeders | |
| --- | --- | --- | --- | --- | --- |
|  | PC1 | PC2 |  | PC1 | PC2 |
| Pasturelands | -0.16 | -4.47 |  | 0.29 | 0.13 |
| Forest | 11.39 | 4.68 |  | 9.98 | 1.00 |
| Non-irrigated crops | -9.37 | 6.42 |  | -3.58 | -7.51 |
| Irrigated crops | -0.56 | -1.13 |  | -0.79 | 0.18 |
| Scrublands | -1.17 | -4.57 |  | -5.94 | 6.19 |
| Permanent crops | 0.00 | -0.01 |  | 0.00 | 0.00 |
| Urban areas | -0.11 | -0.53 |  | 0.04 | 0.00 |
| Bare rocks | -0.03 | -0.41 |  | - | - |
| Others | 0.01 | 0.01 |  | - | - |
| Eigenvalue | 2350 | 1132 |  | 2353 | 1453 |
| Variance (%) | 0.51 | 0.24 |  | 0.54 | 0.35 |


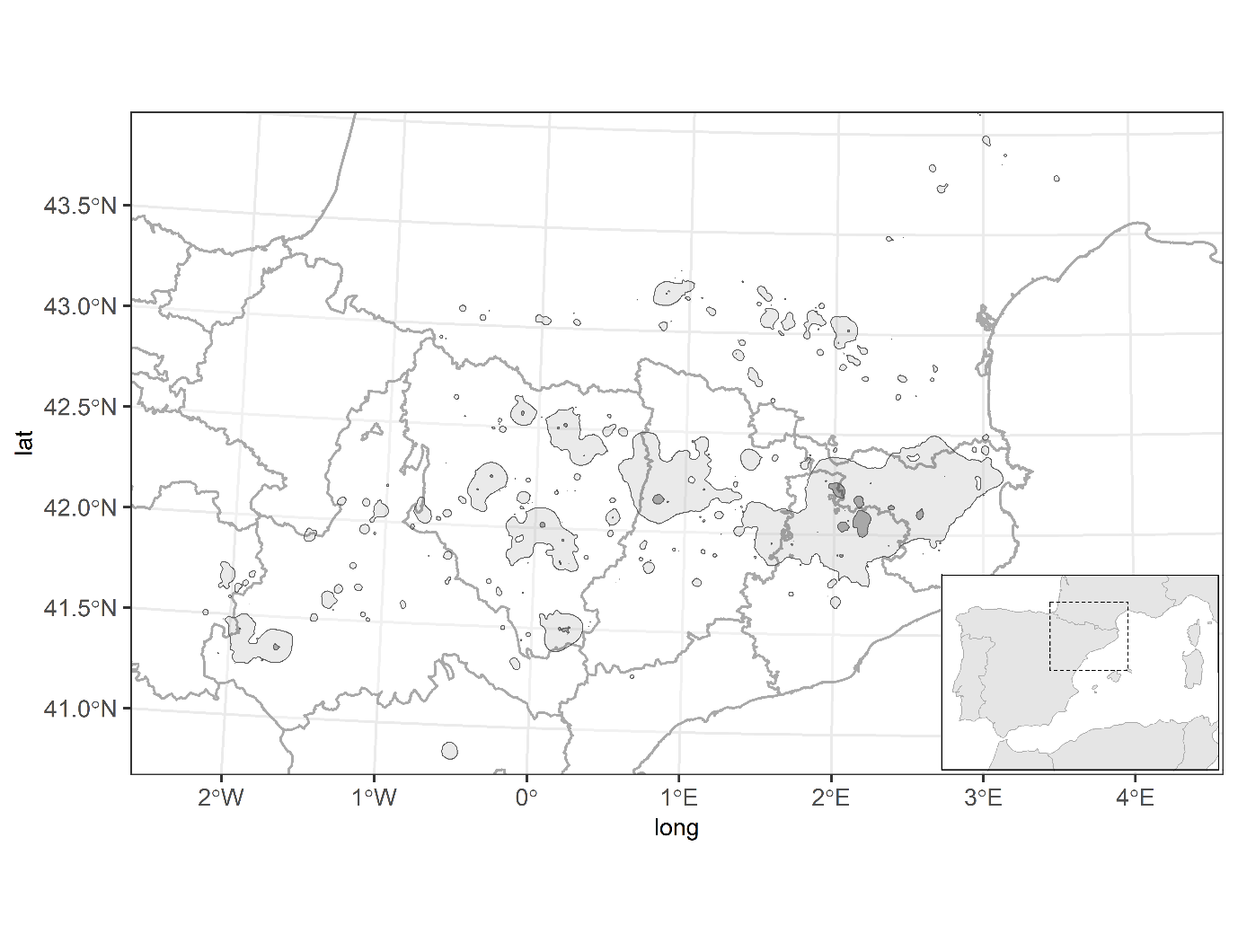
**Figure S1.** Dynamic Brownian Bridge Models home ranges at 50% (dark grey) and 95% (light grey) of 10 non-breeders and 6 breeders of Egyptian vulture tagged in Catalonia (Northeast Spain) at the population level.

**
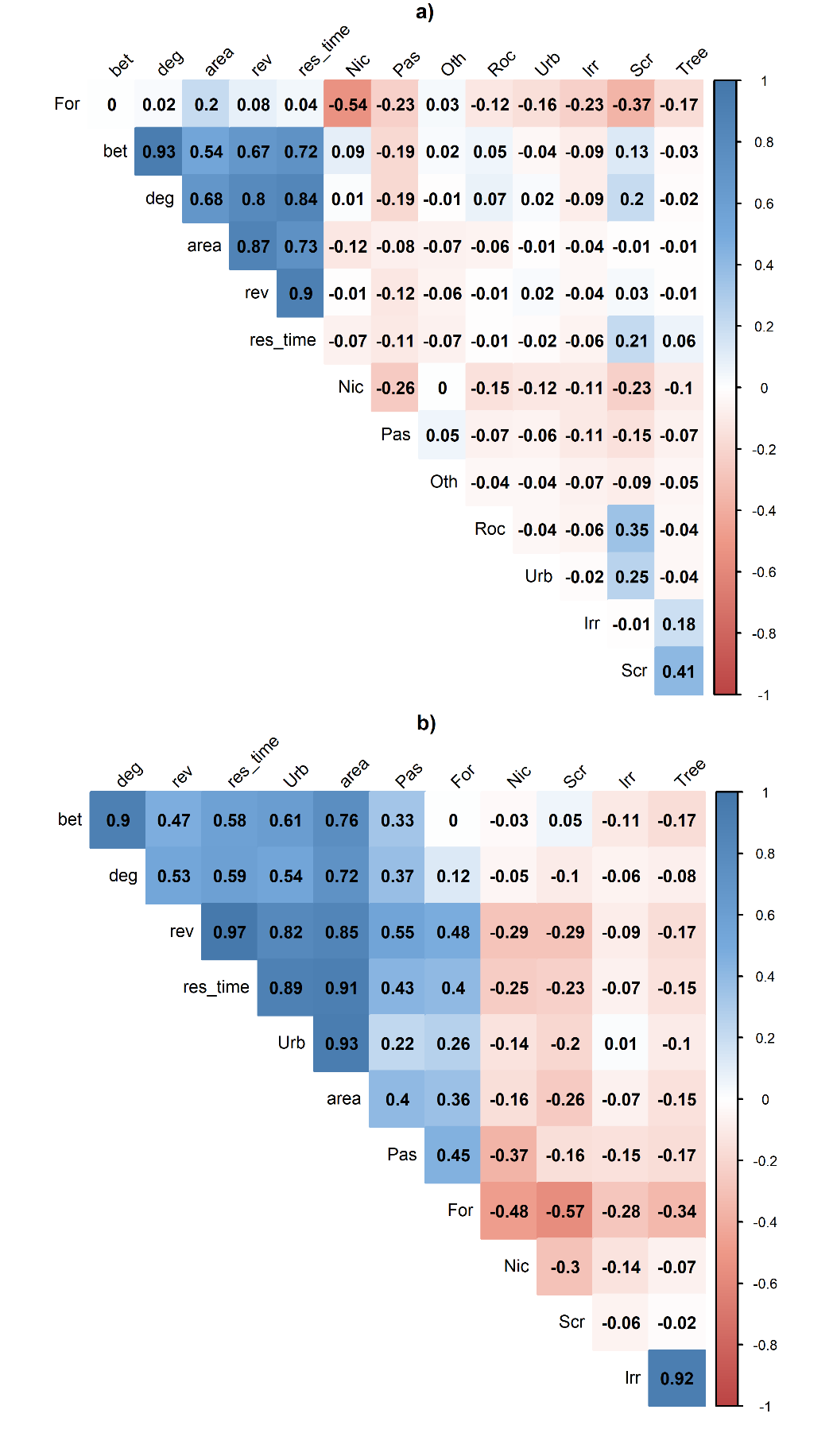
 Figure S2.** Correlation between nodes’ features and the parameters related to node fidelity (number of revisits and accumulated residence time) and local network metrics (*degree* and *betweenness*) for the focal a) non-breeders and b) breeders. Positive correlation is showed in blue, negative correlation in red. bet: *betweennes*. deg: *degree*. area: surface of nodes. rev: number of revisits performs to one specific node. res_time: accumulated residence time on one specific node. For: cover of forest per node. Nic: cover of non-irrigated crops per node. Pas: cover of pasturelands per node. Roc: cover of bare rock per node. Urb: cover of forest per node. Irr: cover of irrigated crops per node, Scr: cover of scrublands per node. Tree: cover of permanent crops per node. Other: cover of other typologies of land uses per node.


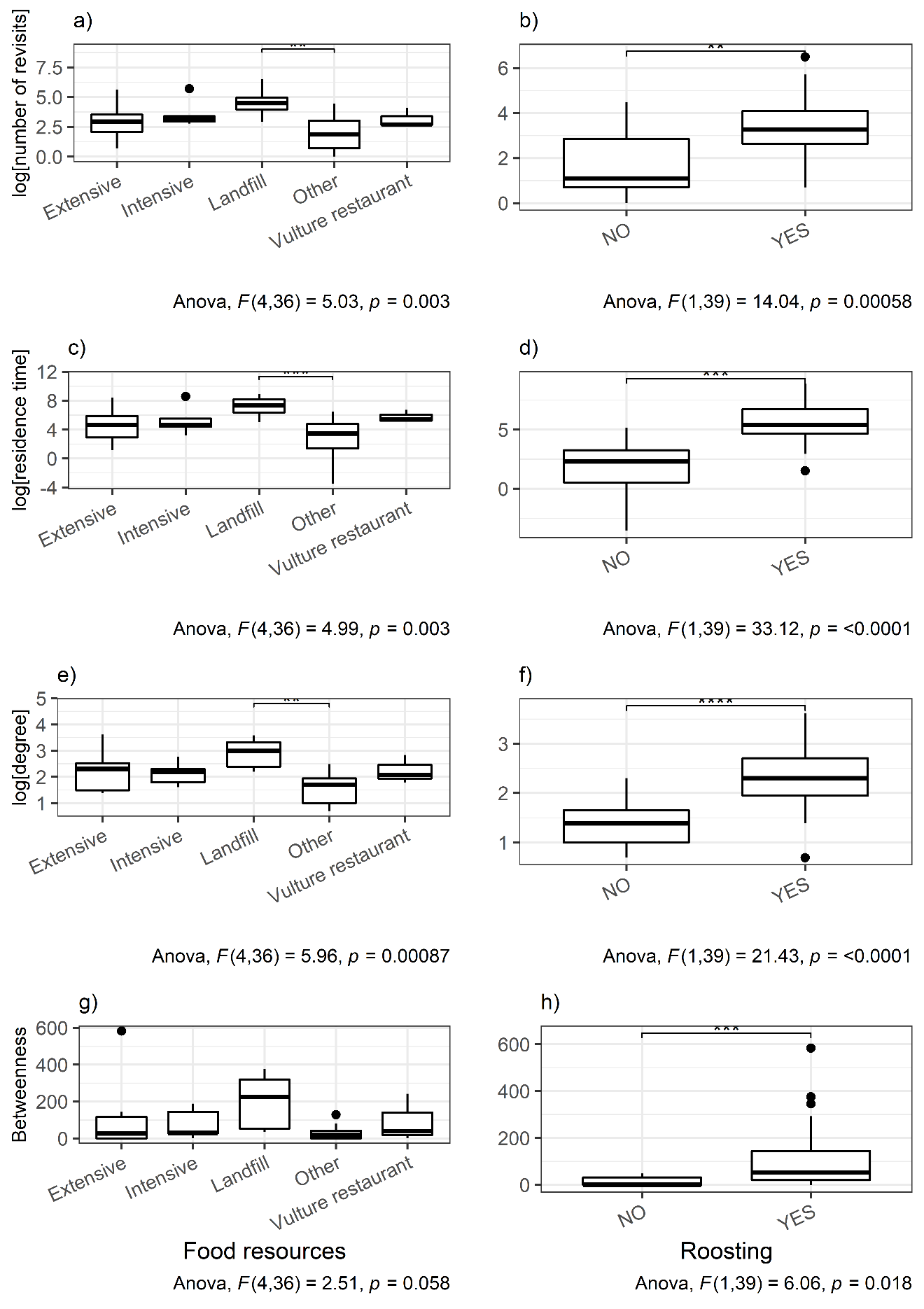


**Figures S3.** Boxplots of non-breeders node fidelity. Boxplots showing the number of revisits (a-b) and the residence time (c-d), and spatial-use networks topology represented by *degree* (e-f) and *betweenness* (g-h) are plotted for non-breeder population. Values are calculated for each node of the non-breeder spatial-use network. ANOVA test was carried out. Aesthetics show significant differences (**P*<0.05, ** *P* <0.01, *** *P* <0.001) with Bonferroni adjustment.


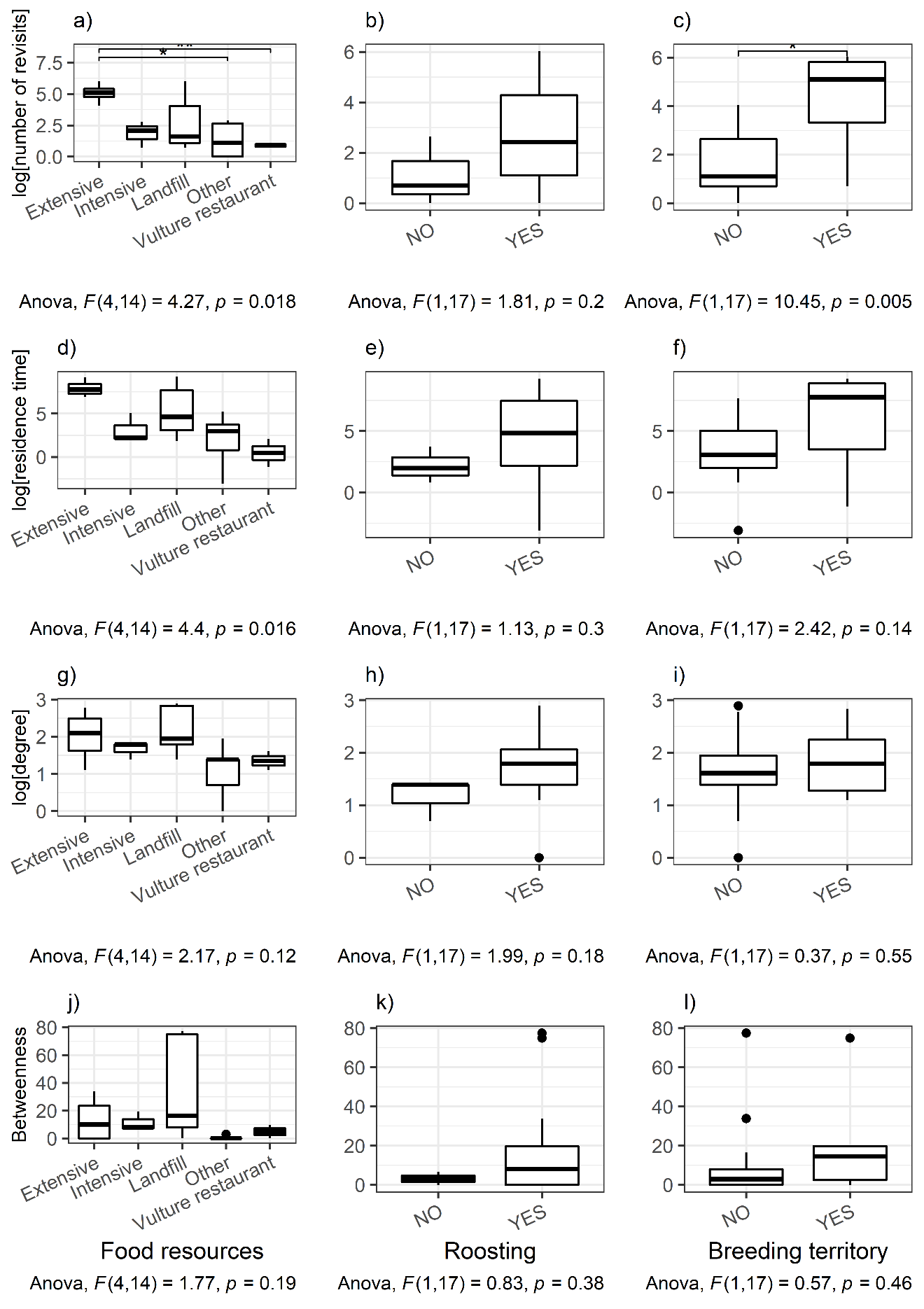


**Figures S4.** Boxplot of breeders nodes fidelity represented by the number of revisits (a-b) and residence time (c-d), and spatial networks topology represented by *degree* (e-f) and *betweenness* (g-h) are plotted for breeder population. Values are calculated for each node of breeder spatial network. ANOVA test was carried out. Aesthetics show significant differences (**P*<0.05, ** *P* <0.01, *** *P* <0.001) with Bonferroni adjustment.
