# Supplementary material for: Resource predictability modulates spatial-use networks in an endangered scavenger species
Source: Mov Ecol. 2023 Apr 20;11:22. doi: 10.1186/s40462-023-00383-4 (PMC10120099; doi:10.1186/s40462-023-00383-4)
Supplement: Supplementary file 2 — Additional file 2: Topology parameters of spatial networks. [file 40462_2023_383_MOESM2_ESM.docx]

**Additional file 2. Topology parameters of spatial networks**

*Degree*

The *Degree* of a node is ‘the number of links connected to a node’ [1]. Sociologists use the ‘six degrees’ theory to demonstrate that we are all well connected. Any person in the world is connected to any another by just six other people (on average) [2]. Ecologically, node *degree* indicates the ‘connectivity’ and refers to how easily one can move to a specific node (‘reachability’, [3]). For example, an animal would tend to return to a node with a high *degree* value and use in a sporadic way the nodes with a low *degree* value.

*Betweenness*

*Betweenness* has been used by authors in several different areas of knowledge (e.g. sociologists, physics and biologists) as ‘the count of how many of geodesic paths between nodes run along each link in the network’ [1]. Geodesic paths refer to ‘the shortest path through the network from one node to another’ [1]. For example, in a simple network of five nodes (A-B-C-D-E), the shortest path between A and B is the one that passes through fewest nodes (e.g. A-C-B) as opposed to any other path from A to B with a greater number of nodes (e.g. A-C-E-D-B). So, *betweenness* is measured by counting the combination of all shortest paths in the spatial network that pass through a particular node. Ecologically, this measure defines which locations are most beneficial in core movements because they occupy a central place in movement networks [3] and so are potentially important areas for conservation and management [4].

**References**

1. Newman MEJ. The Structure and Function of Complex Networks. SIAM review. 2003;45(2):167-256. Available from: <http://www.siam.org/journals/sirev/45-2/42480.html>.
2. Watts D J. Six degrees: The science of a connected age. WW Norton & Company. 2004.
3. Jacoby DMP, Brooks EJ, Croft DP, Sims DW. Developing a deeper understanding of animal movements and spatial dynamics through novel application of network analyses. Methods Ecol Evol. 2012;3(3):574–83.
4. Fortuna MA, Popa-Lisseanu AG, Ibáñez C, Bascompte J. The roosting spatial network of a bird-predator bat. Ecology. 2009;90(4):934–44.
