# Supplementary material for: Resource predictability modulates spatial-use networks in an endangered scavenger species
Source: Mov Ecol. 2023 Apr 20;11:22. doi: 10.1186/s40462-023-00383-4 (PMC10120099; doi:10.1186/s40462-023-00383-4)
Supplement: Supplementary file 3 — Additional file 3: Table S4. Results of the top-ranked models (lowest AIC) for non-breeder and breeder Egyptian vultures accounting for site fidelity (number of revisits and accumulated residence time) and spatial-use network topology (degree and betweenness). [file 40462_2023_383_MOESM3_ESM.docx]

**Additional file 3. Linear regression models**

**Table S4.** Results of the top-ranked models (lowest AIC) for non-breeder and breeder Egyptian vultures accounting for site fidelity (number of revisits and accumulated residence time) and spatial-use network topology (*degree* and *betweenness*). Model **in bold** was selected.

|  | Model | AIC | R2 | *P* |
| --- | --- | --- | --- | --- |
| Non-breeder network | log(number of revisits) ~ PC1 + PC2 + Feeding + Roosting | 128.39 | 0.61 | < 0.001 |
|  | log(number of revisits) ~ PC1 + Feeding + Roosting | 126.64 | 0.61 | < 0.001 |
|  | log(number of revisits) ~ PC2 + Feeding + Roosting | 127.98 | 0.6 | < 0.001 |
|  | **log(number of revisits) ~ Feeding + Roosting** | **126.18** | **0.6** | **< 0.001** |
|  | log(residence time) ~ PC1 + PC2 + Feeding + Roosting | 171.34 | 0.65 | < 0.001 |
|  | **log(residence time) ~ PC1 + Feeding + Roosting** | **169.34** | **0.65** | **< 0.001** |
|  | log(residence time) ~ PC2 + Feeding + Roosting | 173.04 | 0.62 | < 0.001 |
|  | log(residence time) ~ Feeding + Roosting | 171.04 | 0.62 | < 0.001 |
|  | log(degree) ~ PC1 + PC2 + Feeding + Roosting | 72.36 | 0.6 | < 0.001 |
|  | log(degree) ~ PC1 + Feeding + Roosting | 72.33 | 0.58 | < 0.001 |
|  | log(degree) ~ PC2 + Feeding + Roosting | 71.54 | 0.59 | < 0.001 |
|  | **log(degree) ~ Feeding + Roosting** | **71.49** | **0.57** | **< 0.001** |
|  | log(betweenness) ~ PC1 + PC2 + Feeding + Roosting | 475.15 | 0.44 | 0.005 |
|  | log(betweenness) ~ PC1 + Feeding + Roosting | 474.69 | 0.42 | 0.004 |
|  | log(betweenness) ~ PC2 + Feeding + Roosting | 473.36 | 0.44 | 0.002 |
|  | log(betweenness) ~ PC2 + Feeding | 473.12 | 0.42 | 0.002 |
|  | **log(betweenness) ~ Feeding** | **473.12** | **0.42** | **0.002** |
| Breeder network | log(number of revisits) ~ PC1 + PC2 + Feeding + Roosting + Breeding territory | 77.51 | 0.68 | 0.072 |
|  | log(number of revisits) ~ PC1 + Feeding + Roosting + Breeding territory | 75.55 | 0.68 | 0.035 |
|  | log(number of revisits) ~ PC2 + Feeding + Roosting + Breeding territory | 75.51 | 0.68 | 0.035 |
|  | log(number of revisits) ~ Feeding + Roosting + Breeding territory | 73.55 | 0.68 | 0.015 |
|  | **log(number of revisits) ~ Feeding + Breeding territory** | **71.69** | **0.68** | **0.006** |
|  | log(numer of revisits) ~ Breeding territory | 76.24 | 0.38 | 0.004 |
|  | log(residence time) ~ PC1 + PC2 + Feeding + Roosting + Breeding territory | 102.67 | 0.59 | 0.182 |
|  | log(residence time) ~ PC1 + PC2 + Feeding + Breeding territory | 100.67 | 0.59 | 0.104 |
|  | log(residence time) ~ PC1 + Feeding + Breeding territory | 99.86 | 0.57 | 0.072 |
|  | log(residence time) ~ PC2 + Feeding + Breeding territory | 98.69 | 0.59 | 0.054 |
|  | log(residence time) ~ PC2 + Feeding | 96.79 | 0.59 | 0.025 |
|  | **log(residence time) ~ Feeding** | **96.35** | **0.56** | **0.0163** |
|  | log(degree) ~ PC1 + PC2 + Feeding + Roosting + Breeding territory | 50.67 | 0.41 | 0.565 |
|  | log(degree) ~ PC1 + Feeding + Roosting + Breeding territory | 49.27 | 0.39 | 0.468 |
|  | log(degree) ~ PC2 + Feeding + Roosting + Breeding territory | 48.68 | 0.41 | 0.424 |
|  | log(degree) ~ PC2 + Feeding + Breeding territory | 46.87 | 0.41 | 0.302 |
|  | log(degree) ~ PC2 + Feeding | 45.12 | 0.4 | 0.198 |
|  | log(degree) ~ Feeding | 43.6 | 0.38 | 0.125 |
|  | **log(degree) ~ 1** | **44.78** | **-** | **< 0.001** |
|  | log(betweenness) ~ PC1 + PC2 + Feeding + Roosting + Breeding territory | 182.45 | 0.43 | 0.529 |
|  | log(betweenness) ~ PC1 + PC2 + Feeding + Breeding territory | 180.52 | 0.43 | 0.394 |
|  | log(betweenness) ~ PC1 + Feeding + Breeding territory | 179.29 | 0.4 | 0.306 |
|  | log(betweenness) ~ PC2 + Feeding + Breeding territory | 179.49 | 0.4 | 0.325 |
|  | log(betweenness) ~ Feeding + Breeding territory | 178.17 | 0.37 | 0.242 |
|  | log(betweenness) ~ Feeding | 177.29 | 0.33 | 0.191 |
|  | **log(betweenness) ~ 1** | **14.59** | **-** | **0.015** |
