# Supplementary material for: Resource predictability modulates spatial-use networks in an endangered scavenger species
Source: Mov Ecol. 2023 Apr 20;11:22. doi: 10.1186/s40462-023-00383-4 (PMC10120099; doi:10.1186/s40462-023-00383-4)
Supplement: Supplementary file 4 — Additional file 4: Figure S5. Degree (K) and betweenness (B) distributions of spatial-use networks for breeding and non-breeding populations of Egyptian vultures. [file 40462_2023_383_MOESM4_ESM.docx]

**Additional file 4. Distribution of *degree* and *betweenness* define scale-free networks**

In network theory, power law distribution of *degree* (K) and *betweenness* (B) are prospected to evaluate the robustness of a network [1]. As our data are limited, the distribution spans a single order of magnitude and we cannot show whether the underlying distributions for these measures are scale-free, so that the network does show scale-free properties [2]. These analyses were conducted using the “poweRlaw” package [3] in R.


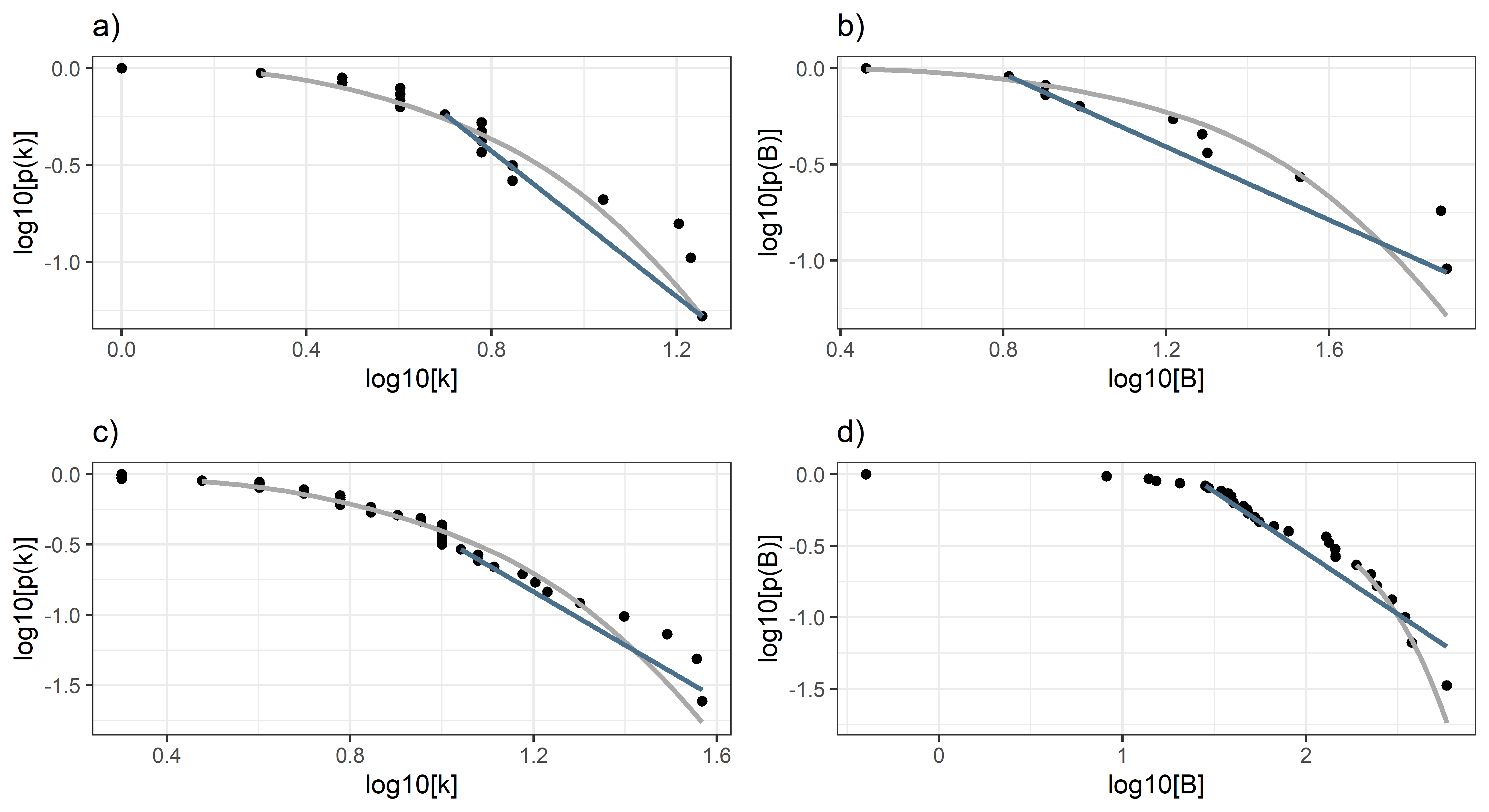


**Figures S5.** *Degree* (K) and *betweenness* (B) distributions of spatial-use networks for breeding and non-breeding populations of Egyptian vultures. Log-log plots of cumulative distribution functions of non-breeders for K (a) and betweenness (b). Log-log plot of cumulative distribution function of breeders for K (c) and betweenness (d). Blue line fits a power law and grey lines fit exponential distribution.

**References**

1. Albert R, Jeong H, Barabási AL. Error and attack tolerance of complex networks. Nature. 2000;406(6794):378–82.
2. Clauset A, Shalizi CR, Newman ME. Power-law distributions in empirical data. SIAM review. 2009;51(4):661-703.
3. Gillespie CS. Package ‘poweRlaw. 2014. Available from: https://cran.r-project.org/web/packages/poweRlaw/index.html.
